# The Germline *SH2B3* ^rs111340708^ Splicing Variant Drives Intron Retention and Protein Instability by Impacting Clinical Outcomes in Core Binding Factor AML

**DOI:** 10.1002/hon.70254

**Published:** 2026-09-12

**Authors:** Marta Rachele Stefanucci, Livia Leuzzi, Linda Montavoci, Davide Paolo Bernasconi, Luca Cimaschi, Alessandra Trojani, Anna Caretti, Lorenzo Del Castello, Roberto Cairoli, Alessandro Beghini

**Affiliations:** ^1^ Department of Health Sciences University of Milano Milano Italy; ^2^ Department of Hematology and Oncology ASST Grande Ospedale Metropolitano Niguarda Milano Italy; ^3^ UOC of Hematology ASST Fatebenefratelli Sacco Milano Italy; ^4^ Bicocca Bioinformatics Biostatistics and Bioimaging Centre University of Milano‐Bicocca Milano Italy

## Abstract

The *SH2B3* gene, also known as *LNK*, encodes an adaptor protein that negatively regulates key hematopoietic signaling pathways, including JAK–STAT, MAPK, and PI3K/AKT, thereby maintaining hematopoietic homeostasis. *SH2B3* interacts with major signaling regulators such as JAK2, MPL, FLT3, and KIT. Loss‐of‐function alterations have been reported in several hematologic malignancies, supporting its role as a leukemia predisposition gene. We previously identified a germline start‐loss mutation (c.3G > A) in *SH2B3* in a family with early‐onset myeloproliferative neoplasm, demonstrating that this variant causes *SH2B3* haploinsufficiency. In the present study, next‐generation sequencing of 149 de novo AML patients identified a frequent intronic polymorphism (rs111340708), located within intron 6 (IVS6) of *SH2B3*. Although this variant has a reported minor allele frequency (MAF) of approximately 12% in European populations, it was enriched in our AML cohort, reaching 34.2% in Core Binding Factor leukemias (CBFLs). The presence of the rs111340708 variant was associated with inferior overall survival, whereas no significant association with progression‐free survival was observed. Functional analyses demonstrated that this polymorphism promotes aberrant IVS6 intron retention in AML cells, resulting in reduced abundance of correctly spliced *SH2B3* transcripts and predicted generation of truncated peptides and/or nonsense‐mediated decay. Consistently, immunoblot analyses of AML patient samples and hematologic cell lines revealed heterogeneous SH2B3 protein expression, including additional SH2B3‐immunoreactive species in variant carriers, together with reduced levels of the canonical SH2B3 protein. Collectively, these findings identify a common germline splicing polymorphism as a novel mechanism contributing to *SH2B3* functional impairment in AML and highlight the potential relevance of non‐coding variants in leukemia pathogenesis, with possible implications for risk stratification and future therapeutic strategies.

## 1 Introduction

Acute myeloid leukemia (AML) is a complex and heterogeneous hematologic malignancy defined by the aberrant proliferation of immature myeloid progenitors. The pathogenesis of the disease stems from a multifaceted interplay between chromosomal aberrations, somatic mutations, and dysregulated signaling cascades that collectively sustain leukemic stem cell (LSC) survival and enforce a block in myeloid differentiation [1, 2, 3]. While the roles of tyrosine kinase receptors, transcription factors and epigenetic modulators are well‐established, there is an increasing appreciation for the contribution of adaptor proteins that fine‐tune intracellular responses to cytokine and growth factor signaling [4]. Among these, *SH2B3* (also known as *LNK*) has emerged as a pivotal tumor suppressor within the hematopoietic compartment [5, 6, 7, 8]. *SH2B3* encodes a member of the SH2B family of adaptor proteins, characterized by a Pleckstrin Homology (PH) domain, a Src Homology 2 (SH2) domain, and an N‐terminal dimerization motif. In stark contrast to its paralogs SH2B1 and SH2B2, which typically potentiate receptor signaling, SH2B3 functions primarily as a negative regulator of tyrosine kinase‐mediated pathways. By binding to phosphorylated receptors and kinases, including JAK2, MPL, FLT3, KIT, PDGFR, TPOR, EPOR it restrains downstream JAK‐STAT, MAPK, and PI3K signaling [9, 10, 11, 12, 13, 14, 15, 16]. Through the attenuation of these proliferative signals, SH2B3 maintains hematopoietic stem cell (HSC) quiescence and prevents aberrant lineage expansion [17]. The link between SH2B3 loss and myeloid transformation was first elucidated in murine models. *Sh2b3*‐deficient mice exhibit hypersensitivity to thrombopoietin and erythropoietin, an expanded HSC pool, and clinical features reminiscent of myeloproliferative neoplasms (MPNs) [18, 19]. These findings are mirrored in clinical observations where somatic *SH2B3* mutations or deletions are identified in subsets of myeloid malignancies, including MPNs and secondary AML [8, 20, 21, 22, 23, 24]. In this context, SH2B3 serves as a molecular “brake,” and its inactivation cooperates with canonical oncogenes such as *JAK2* ^*V617F*^, *FLT3‐ITD*, or mutant *KIT* to accelerate leukemogenesis [7, 20]. Within the landscape of AML, SH2B3 dysregulation plays several distinct pathogenic roles. In patients harboring *FLT3‐ITD*, SH2B3 deficiency amplifies STAT5 activation and enhances leukemic cell proliferation, highlighting a potent synergy between *FLT3* mutations and SH2B3 loss [12]. Similarly, the combination of SH2B3 inactivation and mutant RAS signaling drives aggressive leukemic phenotypes, emphasizing the convergence of cytokine and RAS/MAPK pathways [25]. Critically, loss of SH2B3 is not restricted to secondary AML, its occurrence in *de novo* cases suggests a broader contribution to disease biology [5, 21]. Beyond pathogenesis, *SH2B3* alterations carry significant prognostic and therapeutic implications. Clinically, SH2B3 loss correlates with elevated leukocyte counts, rapid disease progression, and adverse outcomes [5, 17, 21, 25]. Mechanistically, SH2B3 deficiency enhances LSC fitness and self‐renewal, often cooperating with epigenetic lesions such as *DNMT3A* or *TET2* mutations [25]. From a therapeutic perspective, the loss of SH2B3 renders leukemic cells hypersensitive to JAK‐STAT pathway inhibition [11, 24, 26] and SH2B3 expression predicts selinexor sensitivity in acute myeloid leukemia [27]. Preclinical data demonstrate that ruxolitinib or FLT3 inhibitors exert heightened efficacy in SH2B3‐deficient contexts, positioning *SH2B3* status as a potential predictive biomarker for targeted interventions [24, 28]. Our current study demonstrates that the high‐frequency *SH2B3* polymorphism rs111340708 is characterized as a splice region variant located within a repetitive sequence in intron 6 (chr12:111447548–111447572, GRCh38.p14), where it promotes aberrant IVS6 retention in AML, a finding that reflects the wider landscape of splicing instability in this disease [29]. This event introduces a premature stop codon, predicted to generate truncated peptides within the SH2 domain and/or trigger nonsense‐mediated decay (NMD). Supporting this, immunoblot analyses of AML patient samples and hematologic cell lines revealed altered SH2B3 protein architecture accompanied by a marked reduction in functional protein levels. Collectively, these findings identify this common splicing polymorphism as a previously unrecognized mechanism of *SH2B3* inactivation. These data position *SH2B3* as a bona fide tumor suppressor whose inactivation, via mutation, deletion, or post‐transcriptional dysregulation, removes a critical checkpoint on proliferative signaling. Understanding these mechanisms not only advances our insight into AML biology but also offers novel opportunities for precision medicine and the refinement of patient risk stratification.

## 2 Material and Methods

### 2.1 Cell Culture

MOLT‐4 and THP‐1 cells were cultured in RPMI‐1640 supplemented with 10% fetal bovine serum (FBS) and 1% penicillin/streptomycin. HEK293T cells were maintained in DMEM supplemented with 10% FBS, 1% penicillin/streptomycin, and 1% glutamine. All cell lines were cultured at 37°C in a humidified incubator with 5% CO_2_.

### 2.2 Patient Samples

Samples from 149 patients diagnosed with acute myeloid leukemia (AML) were collected at the Department of Hematology, ASST Grande Ospedale Metropolitano Niguarda (Milan), following informed consent and institutional guidelines. Patients were classified into three molecular subgroups: core binding factor AML, including the previously described 32‐patient cohort [30], (CBFL, *n* = 38), non‐CBF AML (*n* = 83), and CEBPA‐mutated AML (*n* = 28). Clinical characteristics are reported in Table 1.

**TABLE 1:** Characteristics of AML patients classified into three different molecular subgroups (CBFL, CEBPA mutated AML and non‐CBFL).

	Patients	Medium age (range)	Female/male	*FLT3* ITD	*FLT3* TKD	*KIT* mut	*JAK2* mut	*CEBPA* m	*CEBPA b*
CBFLs	38	56 (26–84)	14/24	3	6	11	2	3	—
Non‐CBFLs	83	66 (27–96)	46/37	8	11	—	2	—	—
CEBPA mutated AML	28	60 (27–80)	15/13	8	3	—	—	13	15
Total cohort	149	62 (26–96)	75/74	19	20	11	4	16	15

Abbreviations: CEBPA b, biallelic mutation of CEBPA gene; CEBPA m, monoalellic mutation of CEBPA gene; FLT3 ITD, mutation in tandem of FLT3 gene; FLT3 TKD, mutation in tyrosine‐kinase domain of the gene FLT3.

### 2.3 Statistical Analysis

Patients' demographic and genetic characteristics were summarized through descriptive statistics, both considering the overall sample and stratifying for molecular subgroup. Specifically, absolute and relative frequencies are reported for categorical variables whereas for continuous variables the median and range are presented. Survival analyses were conducted to evaluate the Overall Survival (OS) and Progression‐Free Survival (PFS) in the AML CBF cohort. OS was defined as the time elapsed between diagnosis and death, whereas PFS was defined as the time between the achievement of complete remission after induction therapy and either relapse or death, whichever occurred first.

The survival curves for both OS and PFS were estimated with the Kaplan‐Meier estimator, stratifying patients into two groups: those harboring the polymorphism (“SH2B3 group”, *n* = 17) and those without it (“No‐SH2B3 group”, *n* = 15). The Log‐Rank test was performed to assess differences in terms of both OS and PFS among the two groups. The level of significance was set at 5%.

### 2.4 Next‐Generation Sequencing

DNA and RNA were extracted from peripheral blood or bone marrow samples and analyzed using the Ion Torrent Oncomine Myeloid Research Assay (Thermo Fisher Scientific). This targeted panel covers 40 DNA genes and 29 RNA fusion driver genes, including full coding regions of tumor suppressors and hotspot regions of recurrently mutated genes, and is optimized for challenging targets such as CEBPA and FLT3‐ITDs. Libraries were prepared using the Ion Chef System and sequenced on the Ion S5 platform using 400 bp chemistry. Variant calling and annotation were performed using Ion Torrent Suite v5.12.1 and Ion Reporter Software. Minor allele frequencies were assessed after standard quality filtering.

### 2.5 RNA Analysis and End‐Point PCR

Total RNA was isolated from patient samples using a column‐based method and reverse‐transcribed into cDNA (200 ng) using random primers. End‐point PCR amplification of SH2B3 transcripts was performed using gene‐specific primers (Table S1). PCR products were resolved on 1.5% agarose gels and visualized using a ChemiDoc imaging system.

### 2.6 Sanger Sequencing

PCR products generated from SH2B3 cDNA were pooled, purified, and subjected to Sanger sequencing using the same amplification primers. Sequencing was performed on purified fragments (< 300 bp) at a concentration of 15 ng/μL.

### 2.7 Quantitative Real‐Time PCR

Real‐time qPCR was performed using SYBR Green chemistry on 10 ng of cDNA per reaction. SH2B3 expression was normalized to GAPDH using the 2^−ΔΔCt^ method. All reactions were run in triplicate. Primer sequences are listed in Table S1.

### 2.8 Immunoblotting

Total protein was extracted from patient samples and cell lines using RIPA buffer. Proteins were separated by SDS‐PAGE, transferred to nitrocellulose membranes, and probed with antibodies against SH2B3 (rabbit polyclonal, 1:2000, Thermo Fisher Scientific, #PA5‐52154) and GAPDH (mouse monoclonal, 1:10,000, ABclonal, #AC002). Detection was performed using HRP‐conjugated secondary antibodies (anti‐rabbit/anti‐mouse IgG, 1:10,000, Advansta, #R‐05072‐500, #R‐05071‐500) and enhanced chemiluminescence. Signals were acquired on a ChemiDoc imaging system.

### 2.9 Transient Transfection of HEK293T Cells

HEK293T cells were transiently transfected with a human SH2B3 expression construct (SH2B3 Human Tagged ORF Clone, Origene, #RC218359) using FuGENE HD reagent according to the manufacturer's instructions. Cells were harvested 48 hours post‐transfection. Expression of correctly spliced SH2B3 mRNA was assessed by qPCR, with protein expression confirmed by immunoblotting and visualized using a ChemiDoc imaging system. Additional antibody validation using anti‐MYK (mouse monoclonal, 1:2000, Santa Cruz Biotechnology Inc., #sc‐40) is shown in Figure S1.

## 3 Results

### 3.1 Allele Frequency of *SH2B3* ^*rs111340708*^ Intronic Polymorphism in Acute Myeloid Leukemia

**FIGURE 1:**
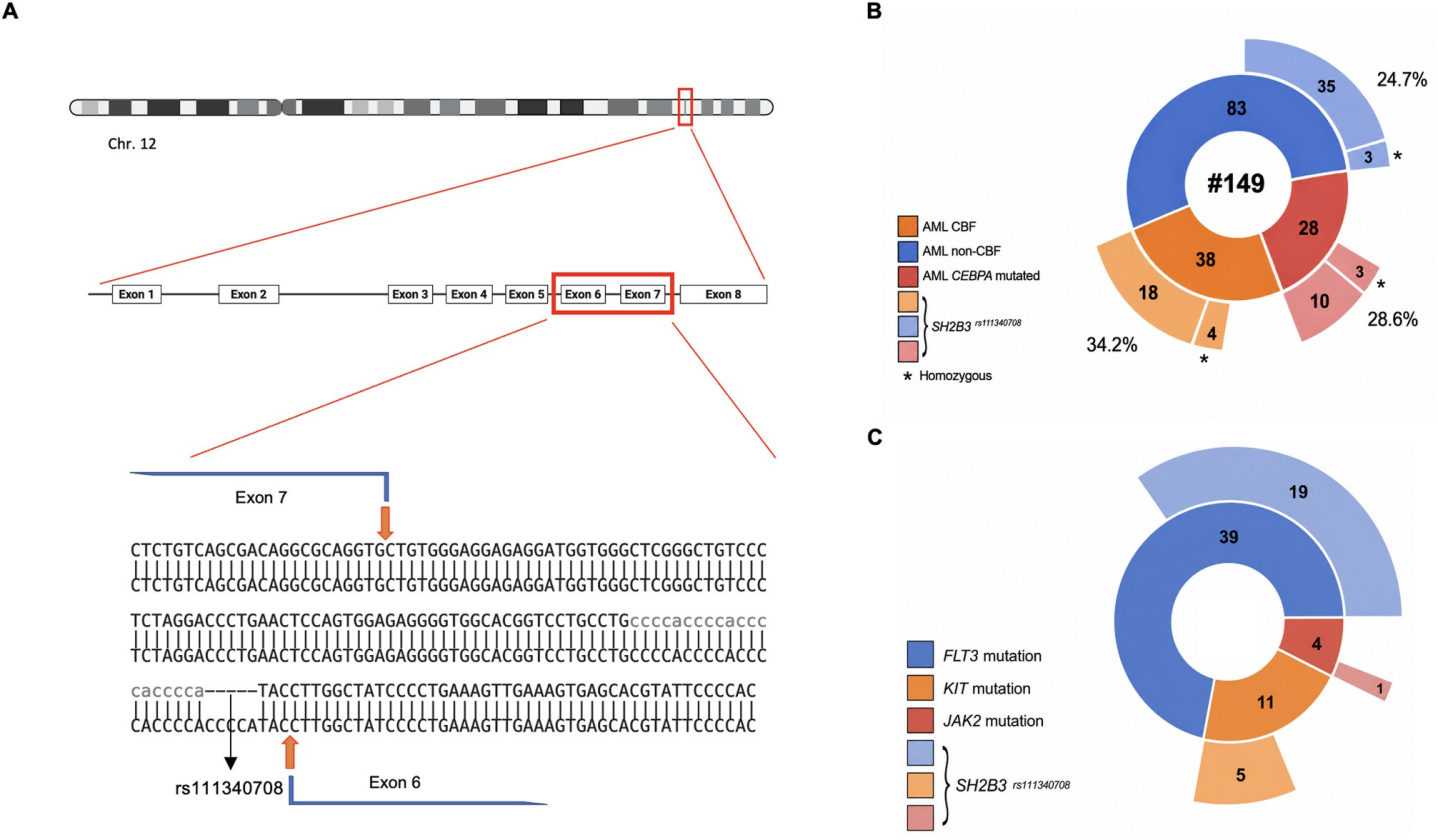
*SH2B3* ^*rs111340708*^ intronic variant. (A) Schematic representation of the human *SH2B3* locus on chromosome 12 and representative analysis of the rs111340708‐associated intron 6 (IVS6) retention event in patient sample #402. (B) Distribution of the *SH2B3* rs111340708 variant in the analyzed AML cohort (*n* = 149), stratified according to molecular subgroups: 22/38 Core Binding Factor (CBF) AML patients (orange), 38/83 non‐CBF AML patients (blue), and 13/28 *CEBPA*‐mutated AML patients (red) carried the variant. (C) Distribution of FLT3, KIT, and JAK2 alterations among AML patients carrying the *SH2B3* rs111340708 variant.

Analysis of the intronic polymorphism *SH2B3* ^*rs111340708*^ (Figure 1A) in a cohort of 149 Acute Myeloid Leukemia (AML) patients revealed a notable deviation from the established European allelic frequency of 12% (ALFA allele frequency https://www.ncbi.nlm.nih.gov/snp/rs111340708#frequency_tab).

The frequency of the polymorphic allele was significantly enriched across all studied AML subgroups. Specifically, the core‐binding factor (CBF) AML group (*n* = 38) exhibited the highest frequency, at 34.2%. Elevated frequencies were also observed in non‐CBF AML (*n* = 83) and CEBPA‐mutated AML (*n* = 28) subgroups, reporting allele frequencies of 24.7% and 28.6%, respectively (Figure 1B). Furthermore, this polymorphic variant was found to be co‐carried with known activating mutations in the *KIT* and *FLT3* genes, suggesting a potential association or functional interplay between the polymorphism and these critical oncogenic drivers in AML (Figure 1C).

### 3.2 Survival Analysis of CBFL Cohort by *SH2B3* ^*rs111340708*^ Status

From the survival analyses focused on the 32 patients with Core Binding Factor (CBF) Acute Myeloid Leukemia (AML) [30], it resulted that the presence of the *SH2B3* ^*rs111340708*^ variant was significantly associated with OS but not with PFS.

**FIGURE 2:**
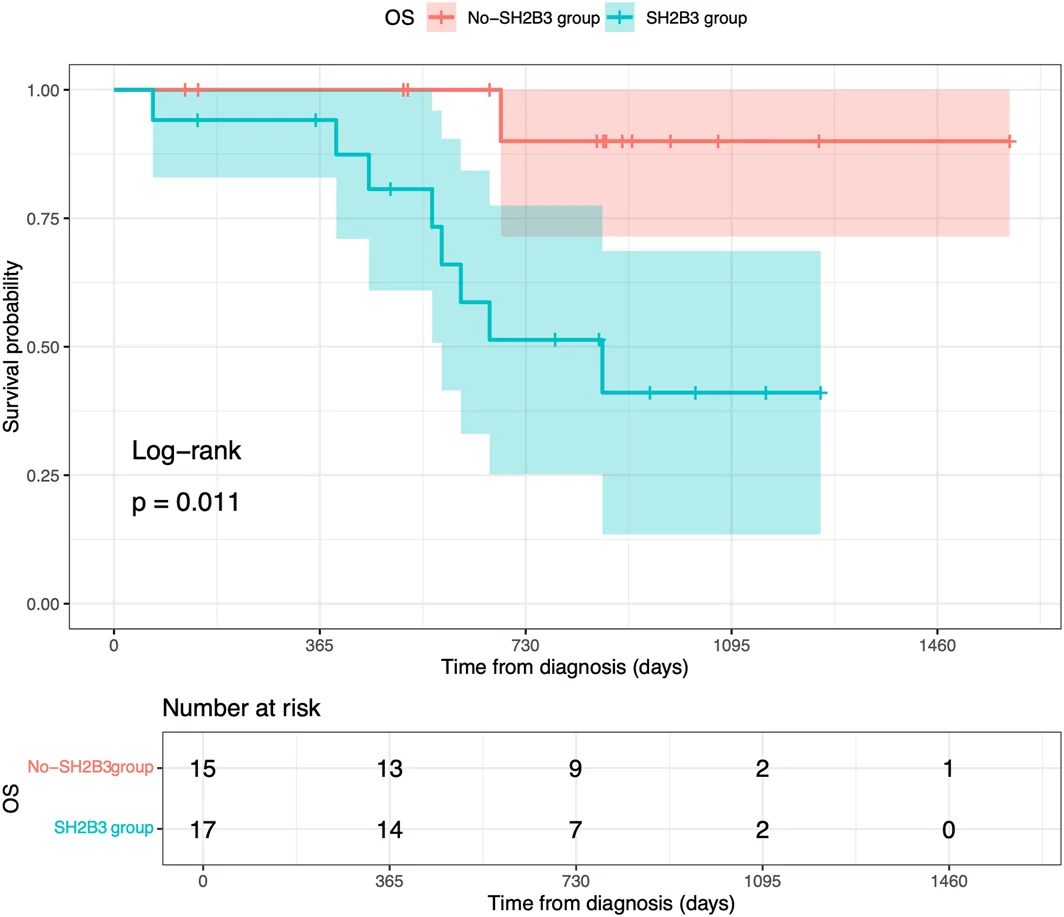
Kaplan‐Meier estimates of overall survival according to *SH2B3* rs111340708 status in CBF‐AML patients. Kaplan‐Meier analysis of overall survival (OS) stratified by *SH2B3* rs111340708 status in CBF‐AML patients. Patients carrying the *SH2B3* variant (blue line) showed significantly reduced overall survival compared with patients without the variant (No‐*SH2B3*, red line) (log‐rank test, *p* = 0.0011). Median OS was 866 days (95% CI, 581–upper limit not estimable) in variant carriers, whereas median survival was not reached in the No‐*SH2B3* group during the observation period.

Specifically, the No‐SH2B3‐mutation group experienced a better overall survival compared to the SH2B3‐mutation group (Figure 2). The median OS for patients with the polymorphism was 866 (95% CI, 581—upper limit not possible to compute) days after the diagnosis, whereas in the No‐SH2B3‐mutation group the median survival time was not reached within the observation period.

**FIGURE 3:**
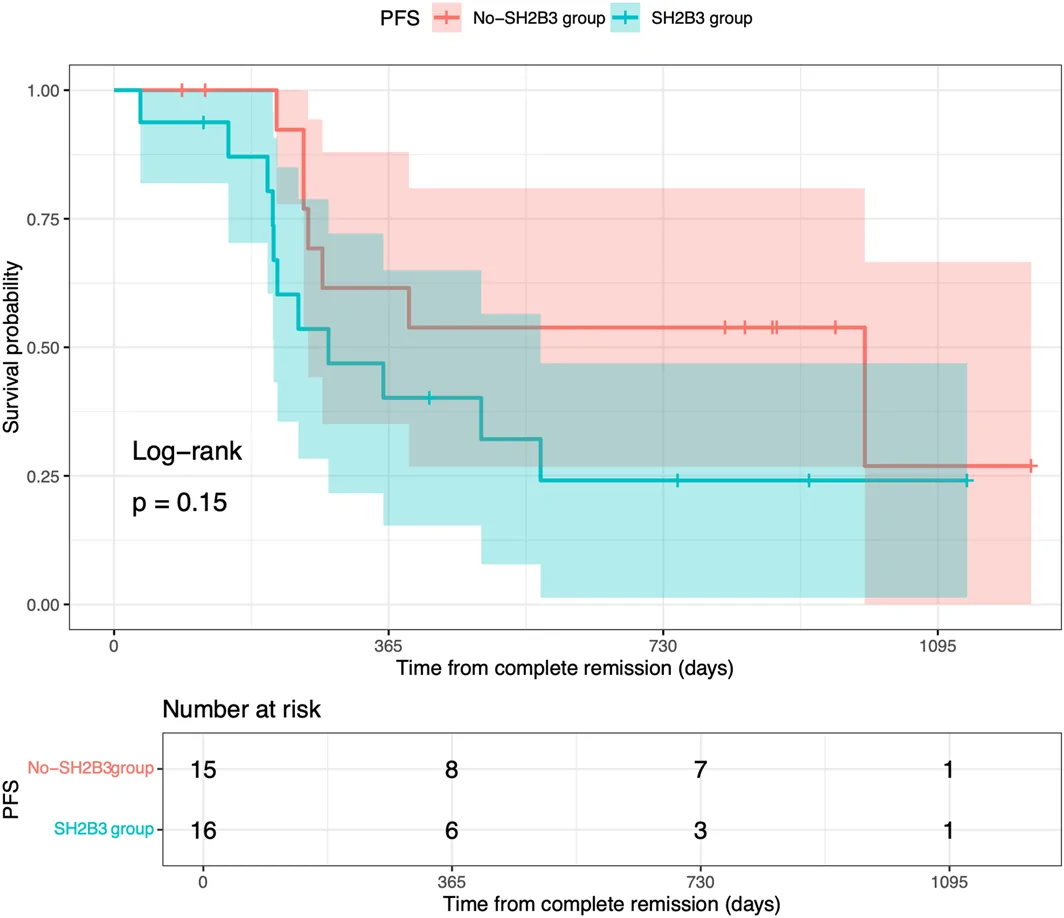
Kaplan‐Meier estimates of progression‐free survival according to *SH2B3* rs111340708 status in CBF‐AML patients. Kaplan‐Meier analysis of progression‐free survival (PFS) stratified by *SH2B3* rs111340708 status in CBF‐AML patients. No significant difference in PFS was observed between patients carrying the *SH2B3* variant (blue line) and those without the variant (No‐*SH2B3*, red line) (log‐rank test, *p* = 0.15). Median PFS was 285 days (95% CI, 212–567) in variant carriers and 998 days (95% CI, 258–upper limit not estimable) in patients without the variant.

Of note, the difference between the two survival curves was statistically significant (Log‐Rank Test, *p* = 0.011). The No‐SH2B3‐mutation group experienced a better survival compared to the other one also in terms of PFS (Figure 3). However, in this case, the association was not statistically significant (Log‐Rank Test, *p* = 0.15). The median survival times experienced respectively by the groups without and with the mutation were 998 (95% CI, 258—upper limit not possible to compute) and 285 (95% CI, 212–567) days.

### 3.3 Transcriptional Impact of rs111340708 on *SH2B3* Splicing in AML

**FIGURE 4:**
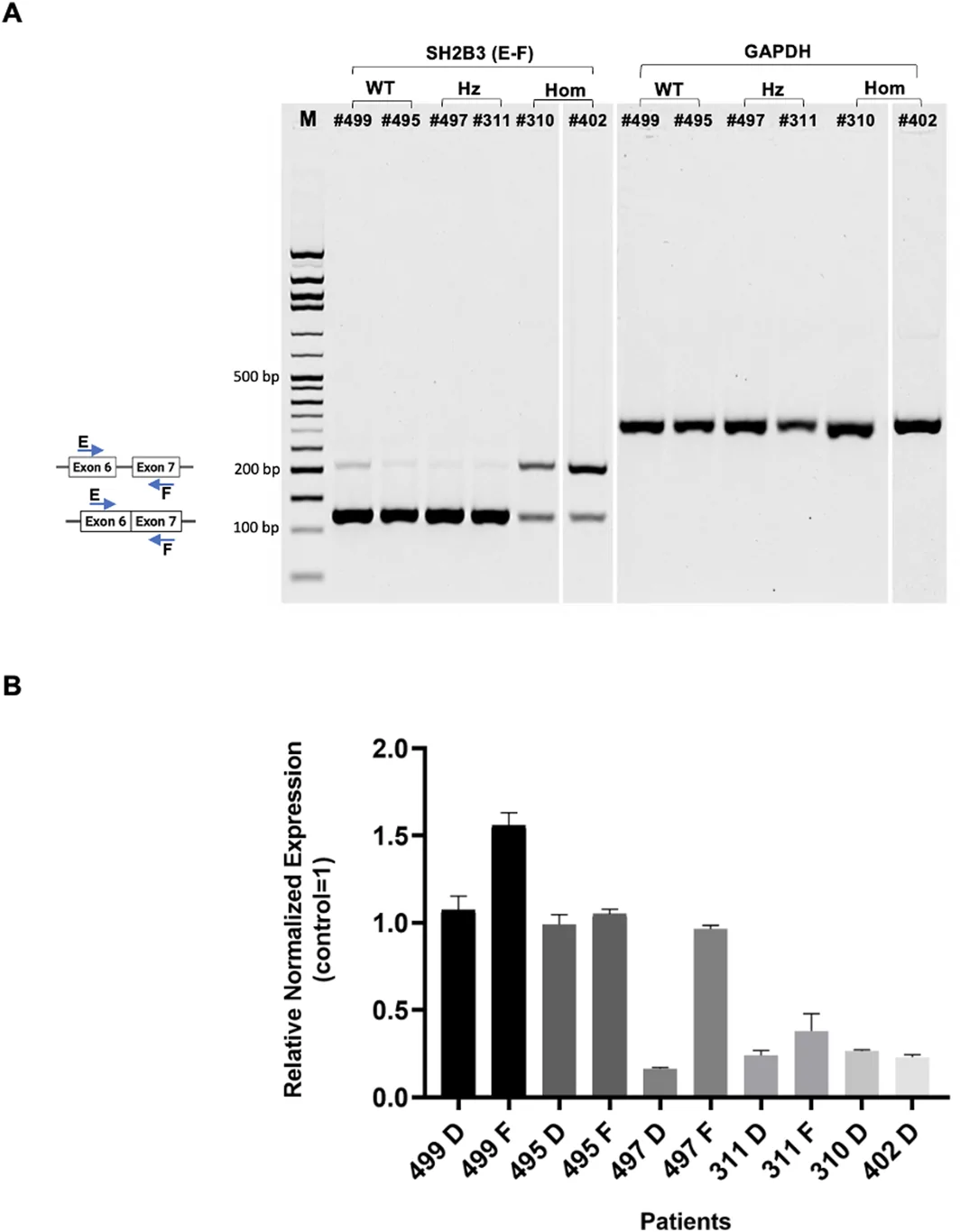
Characterization of *SH2B3* transcript alterations associated with rs111340708. (A) End‐point RT‐PCR analysis of *SH2B3* transcripts using primers spanning exons 6 and 7 in wild‐type (#499, and #495), heterozygous (#311 and #497), and homozygous variant carriers (#310 and #402). The upper band (212 bp) corresponds to the intron 6‐retaining transcript, whereas the lower band (106 bp) represents the correctly spliced transcript. Sequencing of the 212 bp amplicon confirmed the presence of the rs111340708 allele within the retained intronic sequence. GAPDH was used as a loading control. (B) Quantification of correctly spliced *SH2B3* transcripts by RT‐qPCR normalized to GAPDH in AML samples with different *SH2B3* genotypes analyzed at different clinical time points, including diagnosis (D) and follow‐up (F).

To assess the functional consequences of the rs111340708 allelic variant on *SH2B3* pre‐mRNA processing, we performed RT‐PCR analysis comparing transcripts from heterozygous and homozygous variant carriers with those from wild‐type AML patients. Using primers spanning exons 6 and 7, the assay amplified two distinct products: a 106 bp fragment corresponding to the correctly spliced transcript and a 212 bp fragment indicative of intron 6 (IVS6) retention. As expected, wild‐type samples (#499 and #495) predominantly displayed the 106 bp products, consistent with normal *SH2B3* splicing. In contrast, AML samples homozygous for the rs111340708 variant (#310 and #402) showed a marked enrichment of the 212 bp fragments, indicating a major splicing alteration characterized by IVS6 retention. Sequencing of the 212 bp amplicons confirmed that the retained intronic sequence contained the rs111340708 polymorphic allele. This aberrant splicing pattern was also detectable, although to a lesser extent in the competitive end‐point RT‐PCR, in heterozygous carriers (#497 and #311) (Figure 4A). A loading control confirmed comparable RNA quantity and integrity across all samples analyzed. Collectively, these findings demonstrate that the rs111340708 polymorphism promote aberrant *SH2B3* pre‐mRNA splicing, leading to the generation of an intron‐retaining transcript, with a more pronounced effect observed in homozygous variant carriers. To determine whether the rs111340708‐associated splicing defect affected the abundance of correctly spliced *SH2B3* transcripts, we quantified the exon 6–7 spliced product by RT‐qPCR normalized to GAPDH (Figure 4B). This analysis showed reduced levels of correctly spliced *SH2B3* transcripts in homozygous variant samples at diagnosis (#310 and #402) compared with wild‐type samples (#499 and #495). Conversely, longitudinal analysis of heterozygous cases (#497 and #311) revealed an increase in correctly spliced *SH2B3* transcript levels at follow‐up compared with diagnosis (after 143 and 141 days, respectively), suggesting a trend toward increased *SH2B3* expression during follow‐up.

### 3.4 Heterogeneous Protein Expression Associated With the rs111340708 Variant in AML

**FIGURE 5:**
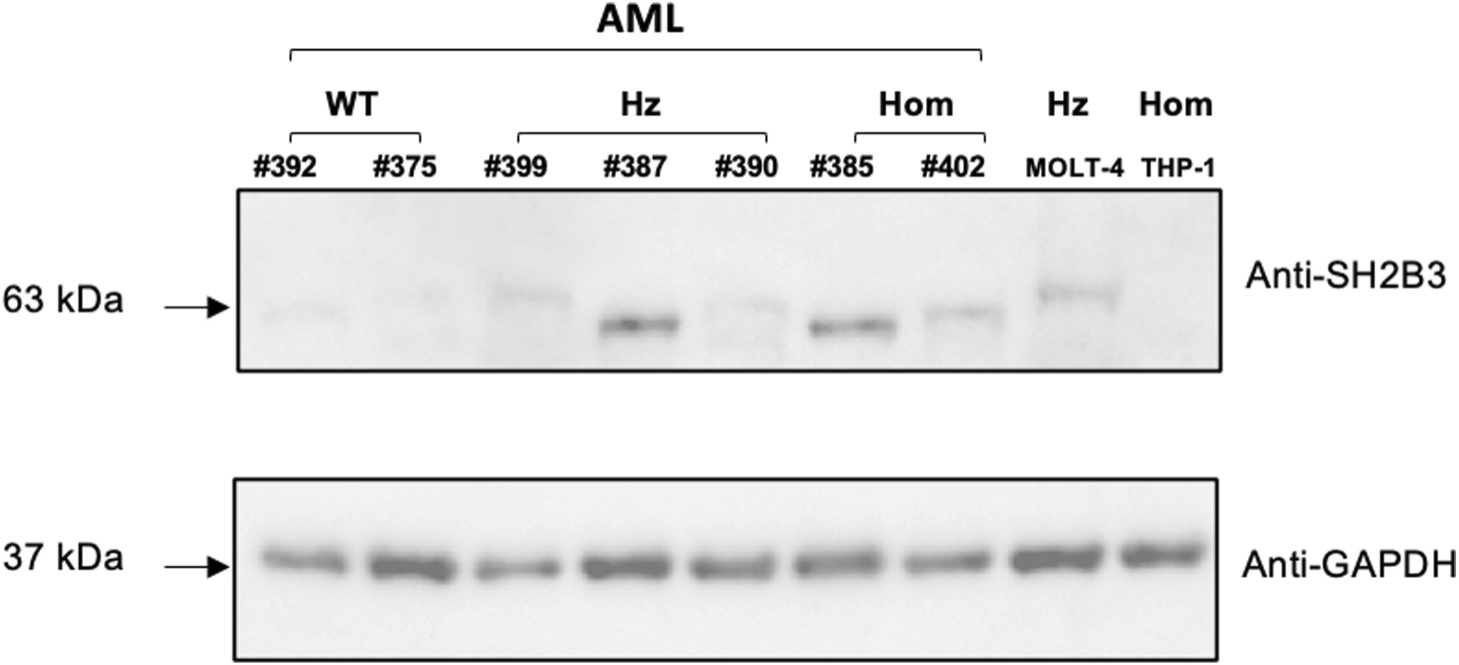
Heterogeneous SH2B3 protein expression in AML primary samples and leukemic cell lines according to rs111340708 variant status. Immunoblot analysis of SH2B3 protein expression in adult primary AML samples (all *CEBPA*‐mutated) and leukemic cell lines carrying different rs111340708 variant configurations. Primary AML samples were classified as wild‐type (WT), heterozygous (Hz), or homozygous variant carriers (Hom) based on genomic characterization. The canonical SH2B3 protein band (∼63 kDa) showed variable intensity among primary AML samples. MOLT‐4 displayed detectable SH2B3 protein expression, whereas THP‐1 showed no detectable SH2B3 signal. Variant status in established cell lines should be interpreted considering possible copy‐number alterations and ploidy abnormalities. Additional SH2B3‐immunoreactive species were observed in selected variant carriers. GAPDH (∼37 kDa) was used as a loading control.

To determine whether the rs111340708‐associated splicing alteration affects SH2B3 protein expression, we evaluated SH2B3 levels by immunoblotting in primary AML samples carrying different rs111340708 genotypes: wild‐type (#392 and #375), heterozygous (#399, #387, and #390), and homozygous variant carriers (#385 and #402). In addition, two leukemic cell lines carrying the rs111340708 variant, MOLT‐4 and THP‐1, were analyzed as complementary models (Figure 5).

Immunoblot analysis revealed the canonical SH2B3 protein band at approximately 63 kDa, with marked inter‐individual variability in expression levels among primary AML samples. Detectable SH2B3 protein expression was observed in samples #387 (heterozygous), #385, and #402 (homozygous), whereas other primary AML samples displayed weak (#392, #399, and #390) or undetectable (#375) signals. Among the leukemic cell lines analyzed, MOLT‐4 showed moderate SH2B3 protein expression, whereas THP‐1 lacked detectable SH2B3 protein.

Because established leukemic cell lines frequently display complex karyotypic alterations, VAF‐based genotype assignment should be interpreted with caution. MOLT‐4 exhibits near‐tetraploid features that may affect the relationship between allelic configuration and conventional zygosity classification. Therefore, variant status in cell lines was interpreted in the context of available genomic characterization.

In addition to the canonical ∼63 kDa SH2B3 band, additional SH2B3‐immunoreactive species were detected in selected variant‐carrying primary samples, particularly #387, #385, #390 and #402. These additional bands may reflect alternative protein products associated with aberrant splicing events, although their precise molecular identity requires further investigation. GAPDH immunodetection confirmed comparable protein loading across all samples. Together with the transcript‐level alterations described above, these findings indicate that the rs111340708 variant is associated with aberrant *SH2B3* splicing and heterogeneous SH2B3 protein expression in AML.

### 3.5 SH2B3 Overexpression in Transfected HEK293T Cells and Validation of Antibody Specificity

To validate the ability of the anti‐SH2B3 antibody to detect exogenous SH2B3 protein and to confirm successful overexpression, HEK293T cells were transiently transfected with a pCMV6‐*SH2B3* ORF construct. Quantitative analysis of *SH2B3* transcripts confirmed robust overexpression in transfected cells compared with non‐transfected controls (Figure S1A). Immunoblot analysis revealed a strong SH2B3‐immunoreactive band in transfected cells, corresponding to the expected molecular weight of the MYC‐tagged SH2B3 protein (∼70 kDa), whereas this signal was absent or markedly reduced in non‐transfected controls (Figure S1B). GAPDH immunodetection confirmed comparable protein loading across samples. In addition, immunoblotting with an anti‐MYC antibody detected a specific MYC‐tag signal exclusively in transfected lysates, confirming the expression of the recombinant MYC‐tagged SH2B3 protein (Figure S1C). Together, these results support the specificity of the anti‐SH2B3 antibody for detection of exogenous SH2B3 and validate the HEK293T overexpression system used for subsequent protein analyses.

## 4 Discussion

In this study, we identify a high frequency intronic *SH2B3* splicing polymorphism (rs111340708) as a previously unrecognized mechanism of *SH2B3* inactivation in acute myeloid leukemia (AML). *SH2B3* (LNK) encodes a critical negative regulator of tyrosine kinase‐mediated signaling, acting as a molecular brake on pathways such as JAK‐STAT, MAPK, and PI3K/AKT to preserve hematopoietic homeostasis [6, 9, 11, 31]. Consistent with this role, loss‐of‐function alterations in *SH2B3* have been implicated in leukemogenesis, where they promote aberrant hematopoietic stem and progenitor cell expansion and cooperate with oncogenic kinases including JAK2^V617F^, FLT3‐ITD, and mutant KIT [12, 14, 18, 20, 32]. Mouse and human studies consistently demonstrate that *SH2B3* deficiency amplifies cytokine signaling and accelerates myeloid transformation, supporting its function as a bona fide tumor suppressor in AML [21, 23, 24, 25, 32].

Our data extend these observations by demonstrating that the rs111340708 polymorphism, although relatively common in the European population (∼12% allele frequency), is markedly enriched in AML, particularly in *CEBPA*‐mutated cases and in core binding factor leukemias (CBFLs), where it reaches an allele frequency of 34.2%. This pattern of enrichment resembles that observed for other germline or inherited variants that confer disease susceptibility or influence leukemic evolution [7, 20]. From the statistical analyses, we observed that patients carrying the variant in the CBFL cohort experienced significantly worse overall survival (OS) than non‐carriers, whereas no significant differences in progression‐free survival (PFS) were detected. These findings raise the possibility that *SH2B3* insufficiency may have a greater impact on late disease behavior or post‐relapse evolution than on the initial response to therapy. One possible explanation is that frontline treatment achieves comparable cytoreduction regardless of *SH2B3* status, whereas impaired negative regulation of cytokine signaling may promote clonal evolution, therapy resistance, or altered leukemic cell fitness following relapse. However, given the limited cohort size (*n* = 32), these observations should be interpreted with caution, as reduced statistical power may have limited the detection of more subtle differences in PFS. Mechanistically, our functional studies demonstrate that rs111340708 intrinsically disrupts *SH2B3* RNA processing by promoting aberrant retention of intron 6 (IVS6). Intron retention predominates in homozygous carriers and is also detectable in heterozygotes, consistent with the broader landscape of splicing dysregulation observed in AML and its association with adverse clinical outcomes [29, 33, 34, 35]. IVS6 retention introduces a premature stop codon predicted either to generate truncated *SH2B3* proteins lacking an intact SH2 domain or to trigger nonsense‐mediated mRNA decay. These transcript‐level alterations are supported by immunoblot analyses showing a marked reduction of the canonical ∼63 kDa SH2B3 protein together with additional immunoreactive bands in variant carriers. These higher‐ and lower‐molecular‐weight species are consistent with the generation of alternative protein products arising from aberrant intron retention, although their precise identity remains to be experimentally validated. Overall, these findings support a post‐transcriptional mechanism contributing to *SH2B3* functional insufficiency.

Longitudinal analyses further suggested a trend toward increased *SH2B3* transcript expression at follow‐up compared with diagnosis in heterozygous variant carriers and, to a lesser extent, in wild‐type patients. Although this observation was independently reproduced in the available longitudinal cases, the number of paired samples remained limited. Moreover, follow‐up bone marrow material from the two patients carrying the homozygous *SH2B3* variant could not be obtained because both experienced disease progression, precluding longitudinal evaluation in this subgroup. Therefore, these findings should be considered exploratory and require validation in larger prospective cohorts. Nevertheless, the concordance between the genomic, transcriptomic, and protein data strongly supports the biological relevance of *SH2B3* splicing dysregulation in AML. The strong enrichment of rs111340708 in CBFLs, which are otherwise characterized by relatively stable genomes, raises the possibility that *SH2B3* insufficiency provides an early or facilitating event during leukemogenesis within this molecular subtype. Notably, population data from the ALFA project indicate striking geographic variability in rs111340708 allele frequency, ranging from near absence in South American populations to very high frequencies in East Asia (https://www.ncbi.nlm.nih.gov/snp/docs/gsr/alfa/). This observation is intriguing in light of reports describing a relatively higher prevalence of CBFL in East Asian populations [36]. However, whether these epidemiological observations are biologically connected remains entirely speculative and warrants investigation in larger ethnically diverse cohorts.

Our findings reinforce and expand previous reports describing *SH2B3* inactivation through somatic mutation, deletion, or transcriptional downregulation in AML [4, 11, 24, 37]. By functionally impairing *SH2B3*, rs111340708 removes a critical inhibitory constraint on cytokine and growth factor signaling in hematopoietic and leukemic progenitors and may cooperate with canonical AML drivers such as FLT3‐ITD, KIT^D816^, RAS/MAPK lesions, and *CEBPA* mutations. The frequent co‐occurrence of rs111340708 with activating FLT3 or KIT alterations in our cohort further supports this cooperative model, consistent with previous evidence demonstrating that *SH2B3* loss amplifies pathological STAT5 activation downstream of these kinases [20]. Finally, our observations may have therapeutic implications. *SH2B3*‐deficient leukemic cells exhibit heightened sensitivity to inhibition of the JAK‐STAT pathway, including JAK inhibitors such as ruxolitinib and FLT3‐ITD inhibitors such as gilteritinib [11, 24, 38, 39, 40]. Although prospective validation is required, *SH2B3* status, whether altered by mutation, deletion, or splicing defects such as rs111340708, may ultimately serve as a predictive biomarker for targeted therapeutic strategies. Moreover, the observed association between rs111340708 and poorer overall survival in our CBFL cohort raises the possibility that this variant may identify a subset of patients at increased risk of unfavorable post‐remission outcomes who could benefit from closer monitoring or alternative post‐remission therapeutic approaches.

In conclusion, we demonstrate that the intronic polymorphism rs111340708 represents a frequent and biologically relevant lesion that disrupts *SH2B3* splicing, reduces functional protein expression, and is likely to cooperate with oncogenic signaling pathways during AML leukemogenesis. These findings expand the spectrum of *SH2B3* alterations contributing to myeloid transformation and further highlight the pathogenic potential of non‐coding variants in AML. As precision therapies targeting tyrosine kinase and JAK‐STAT signaling continue to evolve, *SH2B3* dysfunction may emerge as an actionable vulnerability in genetically defined AML subsets.

## Author Contributions

**Marta Rachele Stefanucci:** methodology, formal analysis, writing – original draft preparation, investigation, data curation. **Livia Leuzzi:** methodology, formal analysis, investigation, data curation. **Linda Montavoci:** methodology, formal analysis. **Davide Paolo Bernasconi:** methodology, validation, formal analysis, investigation. **Luca Cimaschi:** validation, formal analysis, investigation, data curation. **Alessandra Trojani:** methodology, investigation. **Anna Caretti:** methodology, validation, investigation. **Lorenzo Del Castello:** formal analysis, data curation. **Roberto Cairoli:** methodology, resources, supervision, funding acquisition, investigation. **Alessandro Beghini:** conceptualization, methodology, formal analysis, investigation, resources, writing – original draft preparation, supervision, funding acquisition. All authors have read and agreed to the published version of the manuscript.

## Funding

This research was funded by Fondazione Malattie del Sangue Onlus (FMS—Grant No. FMS2024/2025), and the liberal contribution of Dr. Roberto Brusamolino.

## Ethics Statement

The study was conducted in accordance with the Declaration of Helsinki, which ensures ethical considerations for conducting medical research involving human subjects, and approved by the Ethical Committee of “Comitato Etico Milano 3” (code *n*. 224‐052018, 31 May 2018) before initiation.

## Consent

Informed consent was obtained from all subjects involved in the study.

## Conflicts of Interest

The authors declare no conflicts of interest.

## Supporting information


**Figure S1:** Validation of anti‐SH2B3 antibody specificity in HEK293T cells transfected with pCMV6‐SH2B3 ORF construct.

**Table S1:** Primers used for qPCR and PCR end‐point.

## Data Availability

The data that support the findings of this study are available on request from the corresponding author. The data are not publicly available due to privacy or ethical restrictions.
