# Supplementary material for: The Germline SH2B3 rs111340708 Splicing Variant Drives Intron Retention and Protein Instability by Impacting Clinical Outcomes in Core Binding Factor AML
Source: Hematol Oncol. 2026 Sep 12;44(5):e70254. doi: 10.1002/hon.70254 (PMC13570680; doi:10.1002/hon.70254)
Supplement: Supplementary file 1 [file HON-44-e70254-s001.docx]

**Supplementary Figure 1. Validation of anti-SH2B3 antibody specificity in HEK293T cells transfected with pCMV6-SH2B3 ORF construct.**

(A) Quantitative RT-PCR analysis of *SH2B3* mRNA expression in HEK293T cells transfected with the pCMV6-SH2B3 ORF clone (+) compared to untransfected control cells (-). Relative transcript levels were normalized to internal reference controls. (B) Immunoblot analysis using an anti-SH2B3 antibody on whole-cell lysates from transfected (+) and untransfected (-) HEK293T cells. A prominent band corresponding to the recombinant tagged SH2B3 protein (~70 kDa) is detected in transfected cells, whereas no signal is observed in untransfected controls. Lane M denotes the molecular weight marker. GAPDH (~37 kDa) served as a loading control. (C) Immunoblot analysis using an anti-C-MYC antibody confirming expression of the C-terminally MYC-tagged SH2B3 recombinant protein (~70 kDa) exclusively in transfected cells (+). GAPDH (~37 kDa) was used as a loading control. Chemiluminescent signals were captured using the ChemiDoc™ Touch Imaging System.
