# Supplementary material for: The Germline SH2B3 rs111340708 Splicing Variant Drives Intron Retention and Protein Instability by Impacting Clinical Outcomes in Core Binding Factor AML
Source: Hematol Oncol. 2026 Sep 12;44(5):e70254. doi: 10.1002/hon.70254 (PMC13570680; doi:10.1002/hon.70254)
Supplement: Supplementary file 2 [file HON-44-e70254-s002.docx]

| **PRIMER** | **SEQUENCE 5’-3’** |
| --- | --- |
| SH2B3 E Forward | gtg ggg aat acg tgc tca ct |
| SH2B3 F Reverse | gag gga aag tgg agg tgc tg |
| SH2B3 G Forward | ttc agg gga tag cca agc ac |
| SH2B3 H Reverse | ctg gaa gtg gtg gag cat gt |
| GAPDH Forward | aca aca gcc tca aga tca tca g |
| GAPDH Reverse | ggt cca cca ctg aca cgt tg |

**Supplementary** **Table 1**. Primers used for qPCR and PCR end-point.
